# Snub-nosed monkeys (*Rhinopithecus*): potential distribution and its implication for conservation

**DOI:** 10.1007/s10531-018-1507-0

**Published:** 2018-01-23

**Authors:** Jonas Nüchel, Peder Klith Bøcher, Wen Xiao, A-Xing Zhu, Jens-Christian Svenning

**Affiliations:** 1 Section for Ecoinformatics & Biodiversity, Department of Bioscience, Aarhus University, Ny Munkegade 114, 8000 Aarhus, Denmark; 2 Sino-Danish Center for Education and Research, Beijing, 100101 China; 3 Institute of Eastern-Himalaya Biodiversity Research, Dali University, Dali, 671003 Yunnan China; 4 School of Geography, Nanjing Normal University, Nanjing, 210023 Jiangsu China; 5 State Key Laboratory of Resources and Environmental Information System, Institute of Geographical Sciences and Natural Resources Research, Chinese Academy of Sciences, Beijing, 100101 China; 6 Department of Geography, University of Wisconsin-Madison, 550 North Park Street, Madison, WI 53706 USA; 7 Center for Biodiversity Dynamics in a Changing World (BIOCHANGE), Aarhus University, Ny Munkegade 114, 8000 Aarhus, Denmark

**Keywords:** *Rhinopithecus*, Snub-nosed monkey, Species distribution modelling, Historical distribution, Maxent, Conservation

## Abstract

Many threatened species have undergone range retraction, and are confined to small fragmented populations. To increase their survival prospects, it is necessary to find suitable habitat outside their current range, to increase and interconnect populations. Species distribution models may be used to this purpose and can be an important part of the conservation strategies. One pitfall is that such mapping will typically assume that the current distribution represents the optimal habitat, which may not be the case for threatened species. Here, we use maximum entropy modelling (Maxent) and rectilinear bioclimatic envelope modelling with current and historical distribution data, together with the location of protected areas, and environmental and anthropogenic variables, to answer three key questions for the conservation of *Rhinopithecus,* a highly endangered genus of primates consisting of five species of which three are endemic to China, one is endemic to China and Myanmar and one is endemic to Vietnam; Which environmental variables best predict the distribution? To what extent is *Rhinopithecus* living in an anthropogenically truncated niche space? What is the genus’ potential distribution in the region? Mean temperature of coldest and warmest quarter together with annual precipitation and precipitation during the driest quarter were the variables that best explained *Rhinopithecus’* distribution. The historical records were generally in warmer and wetter areas and in lower elevation than the current distribution, strongly suggesting that *Rhinopithecus* today survives in an anthropogenic truncated niche space. There is 305,800–319,325 km^2^ of climatic suitable area within protected areas in China, of which 96,525–100,275 km^2^ and 17,175–17,550 km^2^ have tree cover above 50 and 75%, respectively. The models also show that the area predicted as climatic suitable using Maxent was 72–89% larger when historical records were included. Our results emphasise the importance of considering historical records when assessing restoration potential and show that there is high potential for restoring *Rhinopithecus* to parts of its former range.

**Electronic supplementary material:**

The online version of this article (10.1007/s10531-018-1507-0) contains supplementary material, which is available to authorized users.

## Introduction

Around 28% of mammal species are threatened or near threatened with extinction (IUCN 2014). The main threats are habitat loss and habitat degradation, mainly due to anthropogenic pressure, and many species have had their ranges reduced and are today living in small fragmented populations (IUCN 2008; Schipper et al. 2008). These species are therefore more vulnerable to further anthropogenic impacts, with the small population size also itself constituting a threat, causing vulnerability to inbreeding, demographic stochasticity, diseases and catastrophes (Caughley 1994). One of the main conservation tools is protecting the existing habitat which threatened species inhabit (Joppa and Pfaff 2011; Juffe-Bignoli et al. 2014). However, for species surviving only as small and fragmented populations this may not be sufficient to ensure their survival. Hence, reintroduction may be used to help enhance the long-term survival prospects for these species, by translocation of species to suitable habitat outside its current range, for example previously occupied areas. Additionally, such reintroductions or otherwise assisted range expansions will be necessary to restore the ecological functions of such species (Seddon et al. 2014; Svenning et al. 2016).

Species distribution modelling (SDM) can be used to find potential or suitable habitat outside the species current range, (e.g. Morueta-Holme et al. 2010; Kuemmerle et al. 2011; Chatterjee et al. 2012; Naundrup and Svenning 2015). Many SDMs use only the current distribution of the species and assume that the habitat within represents the best habitat (Braunisch et al. 2008). However, many threatened species have undergone range decline and might be refugee species, i.e., confined to living in suboptimal habitat due to anthropogenic pressures (Kerley et al. 2012). In such cases, SDM, if based on current data from refugee habitats, might misguide conservation management by assuming that remnant populations are found in the most favourable habitat (Cromsigt et al. 2012).

East Asia in general has high biodiversity, e.g. China ranks third in the world in total number of mammal species (IUCN 2008; Smith and Xie 2008), it has a higher diversity of species than either North America or Europe and around one-eighth of all know species on earth (Harkness 1998). Unfortunately, the region also has a high proportion of threatened mammals (Schipper et al. 2008; Hoffmann et al. 2010). China has a long history of anthropogenic impacts on its ecosystems and the impacts have grown drastically in the recent century and especially the last decades, e.g. large areas of forest have been logged during the last half century and the population in China has also more than doubled in the same period (Harkness 1998; Liu and Diamond 2005). This also means that there is a high probability that many of the regions’ threatened species are refugee species, confined to suboptimal habitat. On the other hand, it should be noted that there are large reforestation and afforestation programs going on in China, especially since end of 1990s (Yin et al. 2005; Zhang and Song 2006; Peng et al. 2014; Nüchel and Svenning 2017).

One of the organism groups with many threatened species are primates. Of the 508 known species of primates world-wide, 437 have been assessed by the International Union for Conservation of Nature (IUCN) Red List and of these are more than 66% threatened or near-threatened (IUCN 2016) and the primates in South and Southeast Asia has an even more precarious conservation status, with around 79% threatened with extinction (Schipper et al. 2008). In this study, we focus on one group of East Asian threatened primates, the snub-nosed monkeys (*Rhinopithecus*), comprising five species: *Rhinopithecus bieti* (black (Yunnan) snub-nosed monkey*)*, *R. brelichi* (grey (Guizhou) snub-nosed monkey*)*, *R. roxellana* (golden (Sichuan) snub-nosed monkey*), R. strykeri* (Myanmar (Burmese) snub-nosed monkey*)* and *R. avunculus* (Tonkin snub-nosed monkey). All five species are listed as endangered or critically endangered on the IUCN Red List, with all having decreasing populations. Furthermore, *R. brelichi, R. strykeri* and *R. avunculus* are, with total population sizes under 1000 individuals, on the brink of extinction (Bleisch et al. 2008; Bleisch and Richardson 2008; Xuan Canh et al. 2008; Yongcheng and Richardson 2008; Liu et al. 2009; Xiang et al. 2009; Geissmann et al. 2012). However, while the distribution of *Rhinopithecus* is small and fragmented today, fossil and historical records show that it historically has been widely distributed across South, East and Central China (Kirkpatrick 1995; Li et al. 2002).

The main threats for all five species are habitat loss and hunting (Bleisch et al. 2008; Bleisch and Richardson 2008; Xuan Canh et al. 2008; Yongcheng and Richardson 2008; Geissmann et al. 2012). In addition, future climate changes and increases in human population and resource demand will likely increase pressure on all *Rhinopithecus* populations. All *Rhinopithecus* species therefore face an uncertain future, and to increase their survival chances it is necessary to increase their population size and distributions.

In this study, we use species distribution modelling to identify which climatic factors are associated with the current distribution of *Rhinopithecus* within East Asia. We then assess the extent to which *Rhinopithecus* are refugee species, confined to an anthropogenically truncated niche space (Cromsigt et al. 2012), by incorporating historical records of extirpated populations within the SDM. Finally, to identify areas that may be suitable for reintroduction, we estimate the potential distribution of *Rhinopithecus* within the region, considering climate, habitat availability and the locations of nature reserves.

## Methods

### Study region and distribution data

**Fig. 1:**
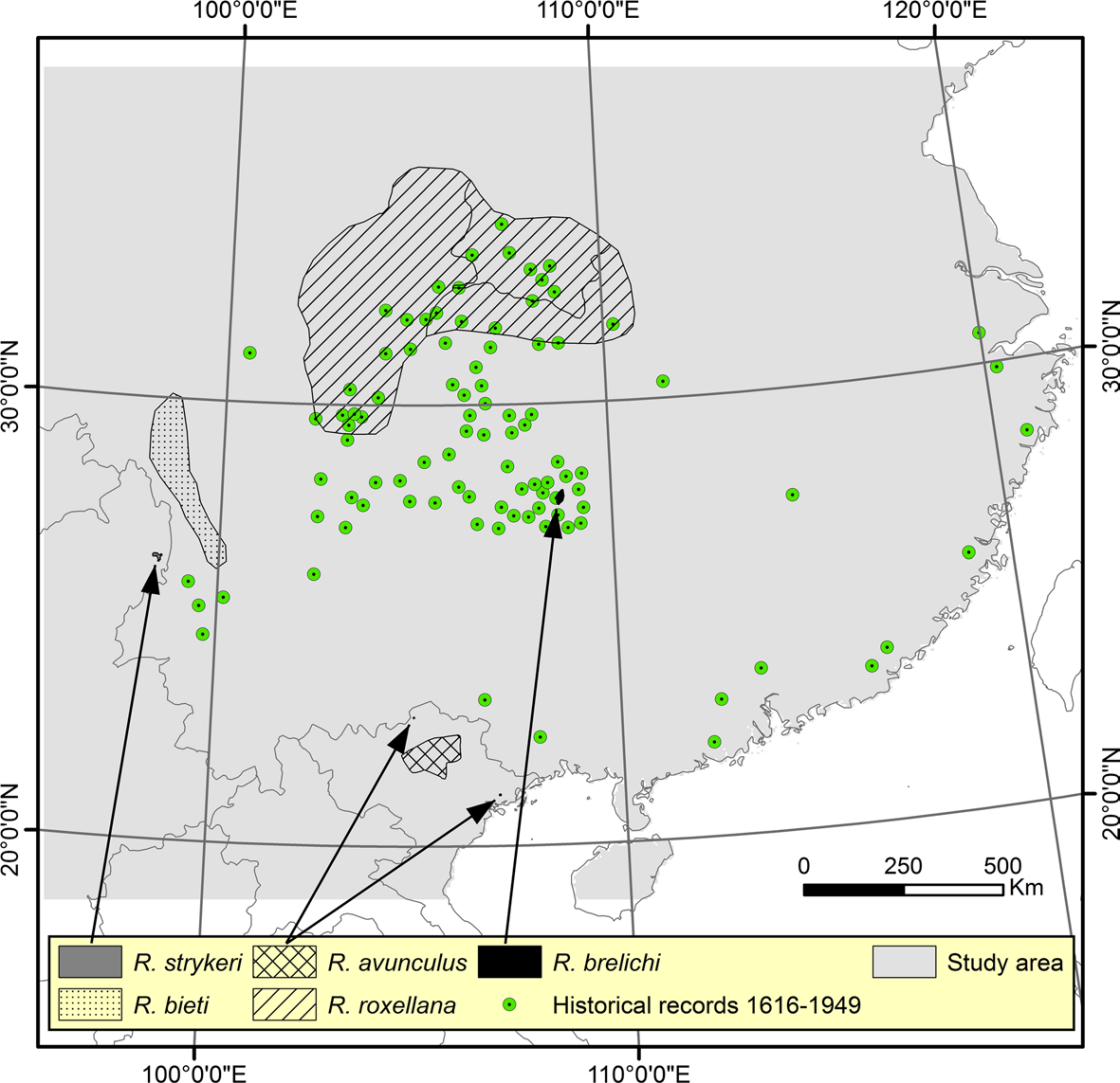
Distribution of *Rhinopithecus*. IUCN range maps for all five species (*R. strykeri, R. avunculus, R. brelichi, R. roxellana & R. bieti*) and historical records from 1616 to 1949

We used range maps from the International Union for Conservation of Nature (IUCN) for all five *Rhinopithecus* species as current distribution data (IUCN 2014). We converted the IUCN polygons to 5 × 5 km grid cells and then to one point per cell (centre of grid). The range estimates were refined by excluding all points outside the species’ known elevation ranges, which are between 200 and 1200 m for *R. avunculus* (Xuan Canh et al. 2008), 570 and 2300 m for *R. brelichi* (Bleisch et al. 2008), 1400 and 2800 m for *R. roxellana* (Yongcheng and Richardson 2008), 1720 and 3190 m for *R. strykeri* (Geissmann et al. 2012), and 3000 and 4700 m for *R. bieti* (Bleisch and Richardson 2008). Furthermore, we also excluded all areas with tree cover below 50%, all areas with human population density above 100 and/or all areas with values above 20 on the human influence index (HII) (see next section for explanation of HII). Afterwards, to reduce spatial bias, we used Occurrence Thinner version 1.04 (Verbruggen 2012; Verbruggen et al. 2013) to thin the points, so we had 142 occurrence points within the IUCN ranges. In addition, historical records for historically extirpated populations of *Rhinopithecus* in China were acquired from Li et al. (2002). By digitalizing maps from Li et al. (2002), we derived 96 approximate locations for historical records from 1616 to 1949, including 70 locations outside the current range of the genus (Fig. 1).

The range data for all *Rhinopithecus* species were combined in most of our modelling (i.e., modelling *Rhinopithecus* as a single taxon), mainly as the historical records could not be identified into species level, but also to overcome niche truncation. All *Rhinopithecus* species, with the exception of *R. avunculus,* occur in subtropical to temperate forest (see Supporting Information Appendix S1 for map of ecoregions/vegetation zones) and hence may be hypothesized to have similar ecological requirements. *Rhinopithecus bieti, R. strykeri, R. brelichi* and *R. roxellana* mainly occur in mixed deciduous and evergreen broadleaf forest, with *R. bieti, R. strykeri,* and *R. roxellana* also occuring in coniferous forest, and *R. avunculus* most deviant, being found in tropical evergreen forest (Bleisch et al. 2008; Bleisch and Richardson 2008; Xuan Canh et al. 2008; Yongcheng and Richardson 2008; Geissmann et al. 2012). Furthermore, *Rhinopithecus* has a high dietary plasticity and occur in areas that have large variations in climate between summer and winter (Long et al. 1994; Yiming 2006; Guo et al. 2007; Xiang et al. 2007b, 2012; Grueter et al. 2009a; Wong et al. 2013). Additionally, we also know that at least some of the species had a more wider distributions as late as within the last 400 years (Li et al. 2002), also in areas that are more climatic different than possibly unoccupied areas within the IUCN polygons. Moreover, as stated in the introduction, some threatened species might be refugee species living in suboptimal habitats (Kerley et al. 2012; Cromsigt et al. 2012) and the current range might not show their full climatic suitable range.

The study area includes a part of East Asia, including areas in China, Myanmar and Vietnam where *Rhinopithecus* are known to occur (Fig. 1). The study area was delimited by drawing a rectangle around a 500-km buffer outside the species range maps and historical occurrence points.

### Environmental and anthropogenic data

Multiple models were calibrated to identify which climate variables are most strongly associated with the distribution of the genus. Initially, eight climatic variables from the WorldClim database (Hijmans et al. 2005) were considered; (1) annual mean temperature (AMT), (2) mean temperature of warmest quarter (MTWQ), (3) mean temperature of coldest quarter (MTCQ), (4) minimum temperature of coldest month (MinTCM), (5) annual precipitation (PANN), (6) precipitation of wettest quarter (PWetQ), (7) precipitation of driest quarter (PDryQ), (8) precipitation of coldest quarter (PColdQ). The four temperature variables, PANN, PDryQ and PWetQ were chosen due to their effect on vegetation and thereby habitat. PColdQ was chosen because the monkeys, except *R. avunculus* and to some degree *R. brelichi*, mainly live in high elevations and precipitation during the coldest quarter will extensively fall as snow and possible limit the food availability.

The historical records for extirpated populations of *Rhinopithecus* were mainly located in Central East (CE) and Southeast (SE) China. We note that the temperature has varied within the period of the historical records (1616–1949) and also subsequently up to the present-day. Generally, the climate was colder in the period of the historical records, with a maximum temperature difference between the coldest period (around 1660 and again around 1840) and current temperature of 1.8 °C for CE China and 1.2 °C for SE China on a decadal time series, and 0.7 °C for CE China and 0.6 °C for SE China on a centennial time series (Ge et al. 2013). To investigate the possible effect of these temperature shifts and, specifically, the colder period during the time of the historical records, we performed a temperature sensitivity analysis by decreasing the current temperature with different magnitudes, subtracting 0.7, 1.5 and 2.0 °C, respectively, from each of the current temperature variables before calibrating the distribution models. The down-adjusted climate variables were used to assess the sensitivity of the models to the different climate and compare the results between models with different adjusted climate data.

Furthermore, we used topographic data from Shuttle Radar Topography Mission (SRTM) (Jarvis et al. 2008) for elevation (ELEV), and computed slope (Slope), standard deviation (STD), and topographic roughness (slope of slope) (TR). Data for protected areas in China were derived from World Database on Protected Areas (IUCN and UNEP-WCMC 2014) and to capture tree cover we used the Moderate Resolution Imaging Spectroradiometer (MODIS) Vegetation Continuous Fields from 2010 (DiMiceli et al. 2011).

We used two different measures of anthropogenic pressure to refine our current distribution data; human population density for 2010 (CIESIN 2015) and the Human Influence Index (HII) (WCS and CIESIN 2005). HII is an index going from 0 (no impact) to 64 (maximum impact) that combines data for population density with data for human land use and accessibility (roads, railroads, navigable rivers and coastlines) and can be used to describe anthropogenic impacts on the environment. The refining was done by excluding all areas within the IUCN range maps with human population density above 100 and/or all areas with values above 20 on the HII.

All data were projected to the Albers Equal Area Conic projection, and converted to their mean values for 5 km × 5 km grid cells. ArcGIS 10.2 (ESRI, Redlands, CA) was used for all GIS operations.

### Maximum entropy modelling/distribution modelling

One of the modelling method used was Maximum entropy modelling (Maxent version 3.3.3k (http://www.cs.princeton.edu/~schapire/maxent/), which is a machine learning method for mapping habitat suitability or estimate the potential distribution of a species (Phillips et al. 2006; Phillips and Dudík 2008; Elith et al. 2011). Maximum entropy modelling is among the best-performing methods for species distribution modelling and frequently outperforms traditional statistical approaches and other species distribution modelling methods (Elith et al. 2006; Phillips et al. 2006).

Pairwise Pearson’s correlation coefficients (r) for all variables over the entire study area were calculated (see Supporting Information Appendix S2 for r values) to quantify collinearity. Although Maxent is relatively robust against collinear variables, collinearity can impair the estimation of the influence of individual variables on the model. Among the climatic variables AMT was highly correlated with MinTCM (r = 0.98), MTWQ (r = 0.92), and MTCQ (r = 0.96). AMT contributed less to the predictive power than MinTCM and MTCQ, and was therefore not included in the modelling. Furthermore, MinTCM and MTCQ were also highly correlated (r = 0.98) and were consequently only included in mutually exclusive models. MTWQ was also correlated with MTCQ (r = 0.78) and MinTCM (r = 0.85). MTWQ did not explain much of the variance, but provided a small increase in predictive power and was therefore kept in four of the final models (Table 1). PWetQ was strongly correlated with PANN (r = 0.94), with PANN contributing more to predictive power than PWetQ; therefore PWetQ was removed. PDryQ and PColdQ were also correlated (r = 0.98) and contributed almost equally to the predictive power. PDryQ indicate the minimum amount of precipitation for a quarter and can be more of an indirect stress factor for vegetation than PColdQ. Therefore, PColdQ was consequently removed.

**Table 1:** The 2 × 4 models using only present distribution data from IUCN (CURR1–4) and also using historical records from 1616 to 1949 (HIST1–4), with variables used and AUC, TSS, AICC and Akaike weight values

Presence data	Model	PA NN	PDryQ	Min TCM	MT CQ	MT WQ	AUC	TSS	AIC_c_	Akaike weight	Threshold
IUCN	CURR1	X	X		X	X	0.900	0.685	3148.094	1	0.320
	CURR2	X	X	X		X	0.896	0.662	3191.302	1.79 × 10^−9^	0.364
	CURR3	X	X		X		0.885	0.601	3207.430	5.62 × 10^−13^	0.323
	CURR4	X	X	X			0.878	0.624	3220.541	1.86 × 10^−16^	0.370
	HIST1	X	X		X	X	0.835	0.576	4800.822	1	0.305
IUCN and historical records	HIST2	X	X	X		X	0.819	0.525	4841.374	2.91 × 10^−9^	0.340
	HIST3	X	X		X		0.801	0.507	4855.961	8.86 × 10^−13^	0.301
	HIST4	X	X	X			0.793	0.515	4886.444	1.16 × 10^−19^	0.330

Out of the four topographic variables, only ELEV contributed a little to the overall predictive power, with the rest having no influence at all. ELEV, STD, Slope and TR were consequently left out of the final models. Both measure for anthropogenic pressure, human population density for 2010 and HII were also left out of our final models, so our final models only model climatic suitability (see Supporting Information Appendix S3, S4 and result section for further).

The final variables in the models were PANN, PDryQ, MTWQ, and MinTCM or MTCQ. This resulted in a total of four models, which were run with current distribution data derived from the IUCN ranges and also with both current distribution data and historical records from 1616 to 1949 (Table 1).

### Model tuning and evaluation

We used area under the curve (AUC) of the receiver operating characteristics (ROC) curve to estimate the predictive power of our models. The maximum AUC value is 1, achieved by perfect discrimination between occupied and non-occupied cells, while a model with no better predictive ability than random choice will result in an AUC value of 0.5. In practice, models with an AUC above 0.75 are considered potentially useful (Phillips and Dudík 2008). We note that for presence-only data, like in the present study, the highest achievable AUC is < 1 (Phillips et al. 2006).

AUC values may not always be the optimal method to evaluate model performance, e.g. as AUC weighs omission and commission errors equally, and the geographical extent to which models are carried out in can highly influences the AUC values (Lobo et al. 2008). Therefore, the models were also evaluated using the true skill statistic (TSS), which in contrast to kappa is independent of prevalence (Allouche et al. 2006). TSS uses the same scale as kappa and has values between 0 and 1, where 0–0.4 = poor, 0.4–0.5 = fair, 0.5–0.7 = good. 0.7–0.85 = very good, 0.85–0.9 = excellent and 0.9–1 = perfect.

In addition to TSS and AUC, we used the R package ENMeval version 0.1.1 (Muscarella et al. 2014) to tune the features and regularization multiplier settings in Maxent, and ENMTools version 1.4.4 (Warren et al. 2008, 2010) to calculate the sample size corrected Akaike Information Criterion (AIC_C_) and Akaike weights value for our models. AIC_C_ has been showed to outperform AUC-based methods for model selection in many cases (Warren and Seifert 2010). The final models were run using default settings, except the regularization multiplier value, which was set to 2.5, maximum iterations was changed to 5,000 and all features were used. To derive suitability and predictive maps the final models were run 10 times with cross validate as replicated run type.

### Suitable habitat and potential distribution

Our predictive presence-absence map was derived using the 10th percentile training presence thresholds, which selects the value above which 90% of the training samples are correctly classified. Selection of the best thresholds can be difficult and depends on the sample size and the purpose of the study (Pearson et al. 2004; Liu et al. 2005; Freeman and Moisen 2008; Bean et al. 2012), but in general maximum training sensitivity and specificity, and equal training and specificity perform best (Jiménez-Valverde and Lobo 2007; Liu et al. 2013; Cao et al. 2013). The above thresholds are more conservative thresholds than the minimum training presence threshold, which correctly predicts every training sample (See Supporting Information Appendix S5 for a comparison of thresholds effect on our predictive presence-absence map). As an alternative, which is better protected against overfitting, we also generated a rectilinear bioclimatic envelope model, defined as areas within minimum and maximum values of the five climatic variables, PANN, PDryQ, MinTCM, MTCQ and MTWQ, using only current distribution data derived from IUCN ranges and using both current distribution data and historical records, in ArcGIS. We did this for all species combined and also for each species separately. The latter was done to compare the species- and genus-level results, mainly for checking their consistency. Furthermore, the models with current and historical distribution data were modelled using both current climate data and current climate data adjusted with − 0.7, − 1.5, and − 2.0, respectively, to provide estimates accounting for the cooler climate during the period that the historical records represent.

To assess how much of the climatic suitable area includes tree cover above a certain percentage and were within protected areas, the outputs from the rectilinear bioclimatic envelope models were overlaid with tree cover, derived from Moderate Resolution Imaging Spectroradiometer (MODIS) Vegetation Continuous Fields from 2010 (DiMiceli et al. 2011), and protected areas in China (IUCN and UNEP-WCMC 2014).

## Results

The four models with current distribution data derived from IUCN ranges (CURR1-4, Table 1) resulted in AUC values, between 0.878 and 0.900 and TSS values between 0.601 and 0.685, indicating good predictive power. The four models with both current distribution data and historical records (HIST1-4, Table 1) resulted in AUC values, between 0.793 and 0.835 and TSS values between 0.507 and 0.576, again indicating good predictive power. AIC_C_ and the derived Akaike weight scored the models with the variables PANN, PDryQ, MTCQ and MTWQ, as the best models for both current data only (CURR1) as well as for current and historical data together (HIST1). For all models, the ranks of the models based on AIC_C_ and Akaike weights were consistent with the ranks/values of AUC and to some degree also the values of TSS (Table 1).

Human population density for 2010 contributed to our the predictive power of our models, whereas HII contributed only sligthly to the predictive power of our models (see Suporting Information Appendix S3). The response curves from the Maxent model indicate that there is a negative relationship between the current distribution of snub-nosed monkeys and both of the anthropogenic variables, as the probability of presence in the Maxent prediction decreased with increasing population density and HII (see Suporting Information Appendix S3).

**Fig. 2:**
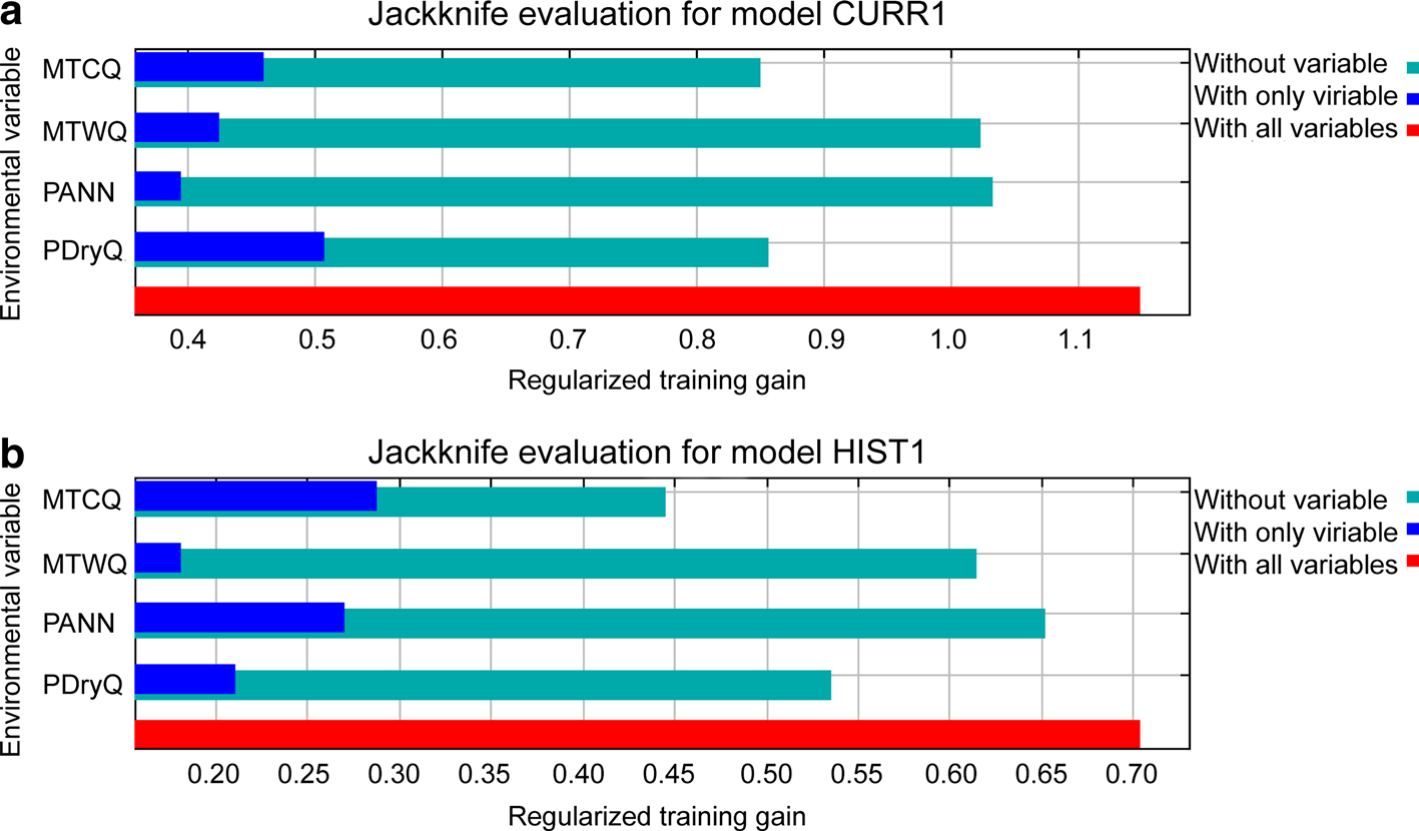
Results of jackknife evaluation of the relative importance of the variables with respect to regularized training gain for (**a**) model CURR1 and (**b**) model HIST1. For acronyms see the environmental data section in methods

PANN, MTCQ or MinTCM, PDryQ and MTWQ were the variables that best explained the distribution of *Rhinopithecus* (all five species modelled together). This was the case when the distribution was modelled using current distribution data (derived from IUCN ranges) as well as both current distribution data and historical records. However, the variables importance changes a little between model CURR1 and HIST1. For model CURR1, using only current distribution data, the Maxent jackknife evaluation indicate that PDryQ, followed by MTCQ and MTWQ were the strongest predictors by themselves. For model HIST1, using historical data in addition to current distribution data, MTCQ, followed by PANN and PDryQ were the strongest predictors. In both models, MTCQ was the variable that decreases the gain the most when it was omitted, which indicate that it has the most information that is not explained by the other variables (Fig. 2).

**Fig. 3:**
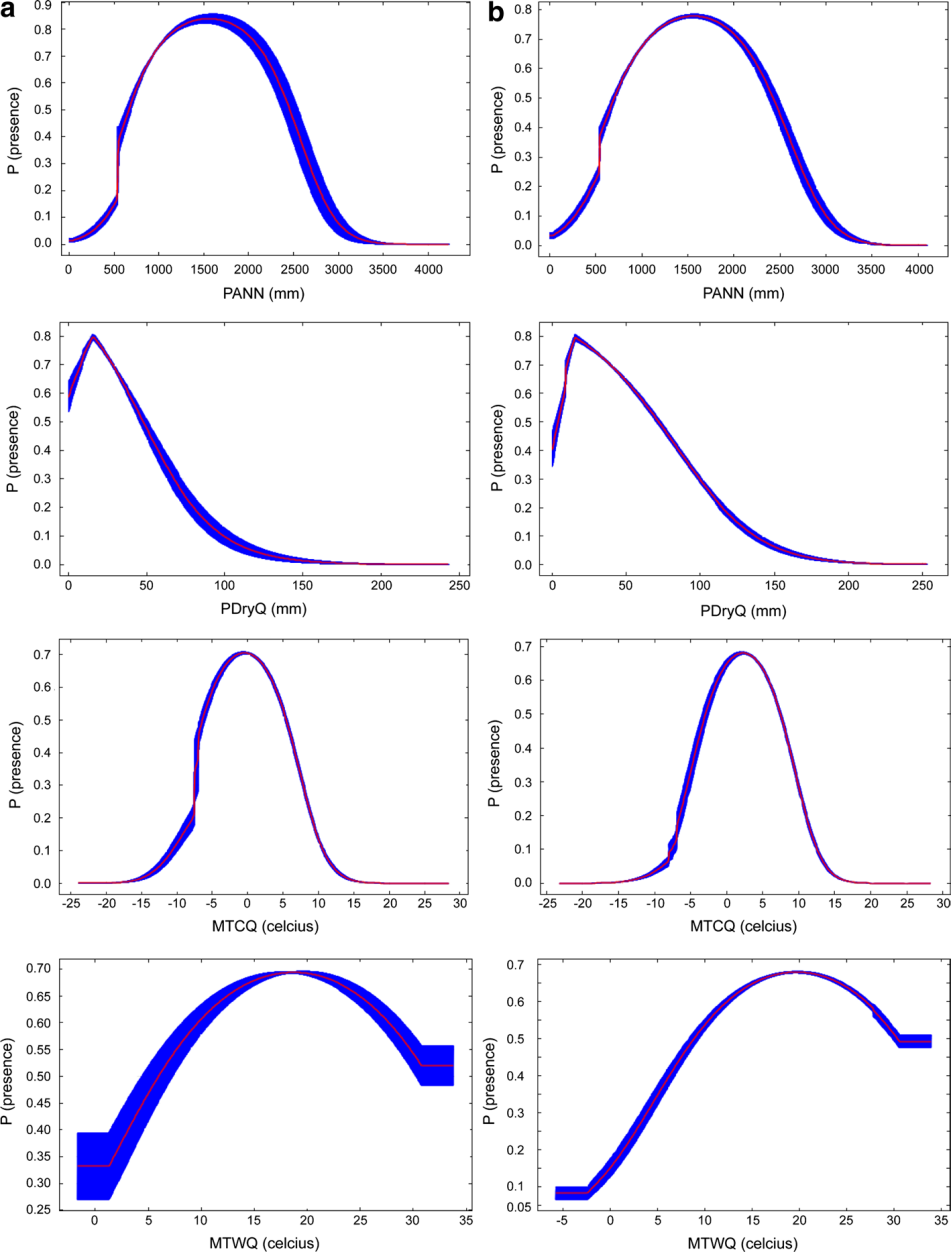
Estimated response curves (logistic output: probability of presence), which show how the logistic prediction changes as each environmental variable is varied, keeping all other environmental variables at their average sample value, for (**a**) model CURR1 and (**b**) model HIST1. For acronyms see the environmental data section in methods

**Fig. 4:**
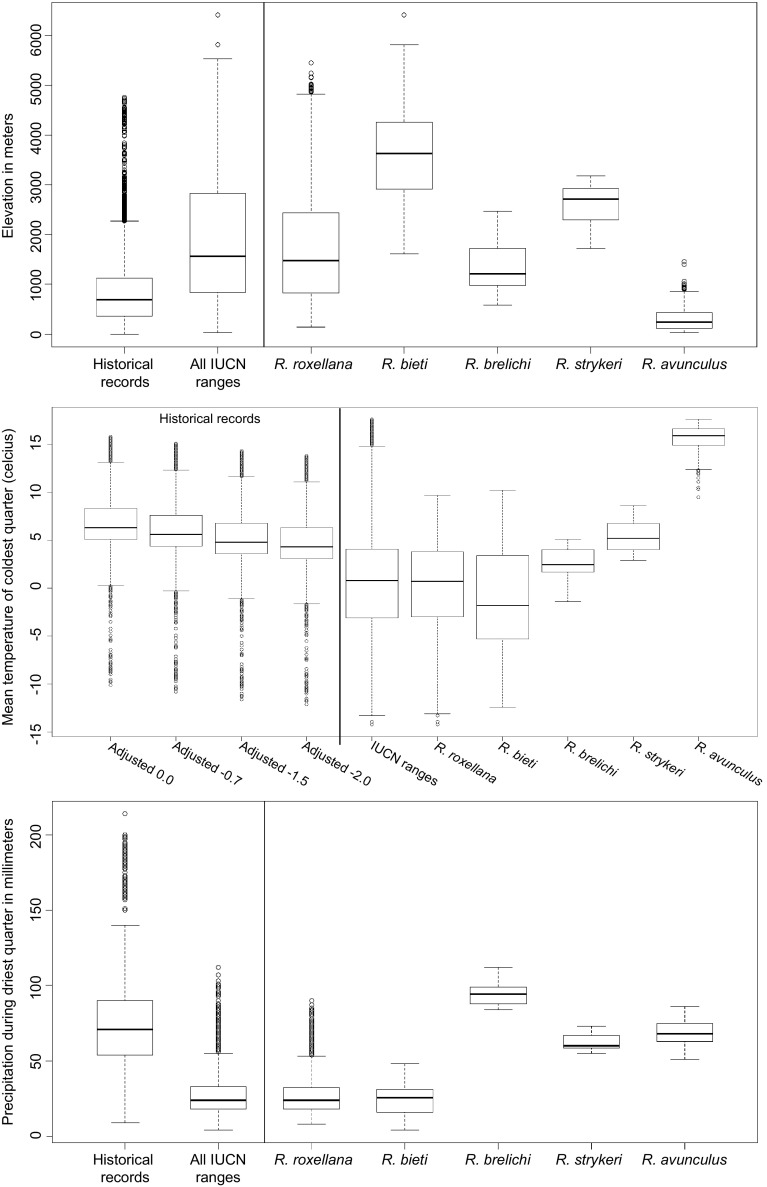
Boxplot of elevation (ELEV), mean temperature of coldest quarter (MTCQ) and precipitation during driest quarter (PDryQ) for the historical records, the IUCN ranges (all five species together) and for the five species separately. See Supplementary Information Appendix S3 for boxplot of PANN, MinTCM and MTWQ. MTCQ for the historical records is divided into four categories; current climate data without adjustments, current climate data adjusted with − 0.7, − 1.5, and − 2.0 °C, respectively

The estimated responses curves in Maxent had the same tendencies between model CURR1 and HIST1, but with higher mean values for PANN, PDryQ, and MTCQ when historical records were also included (Fig. 3). Consistent with the SDM results, the range of the different environmental variables varied between the current distribution and the historical records: All species, except *R. avunculus,* today generally occur in colder areas and higher elevation than the historical records. This is also the case if the models are calibrated using the down-adjusted temperature data to better match the historical climate reference (Fig. 4, and Supporting Information Appendix S6). *Rhinopithecus roxellana* and *R. bieti* also generally occur in drier areas then the historical records (Fig. 4, and Supporting Information Appendix S6). The medians for all climatic variables and elevation differed between the current distribution and the historical records (Mann-Whitney *U* tests, p < 0.0001).

**Fig. 5:**
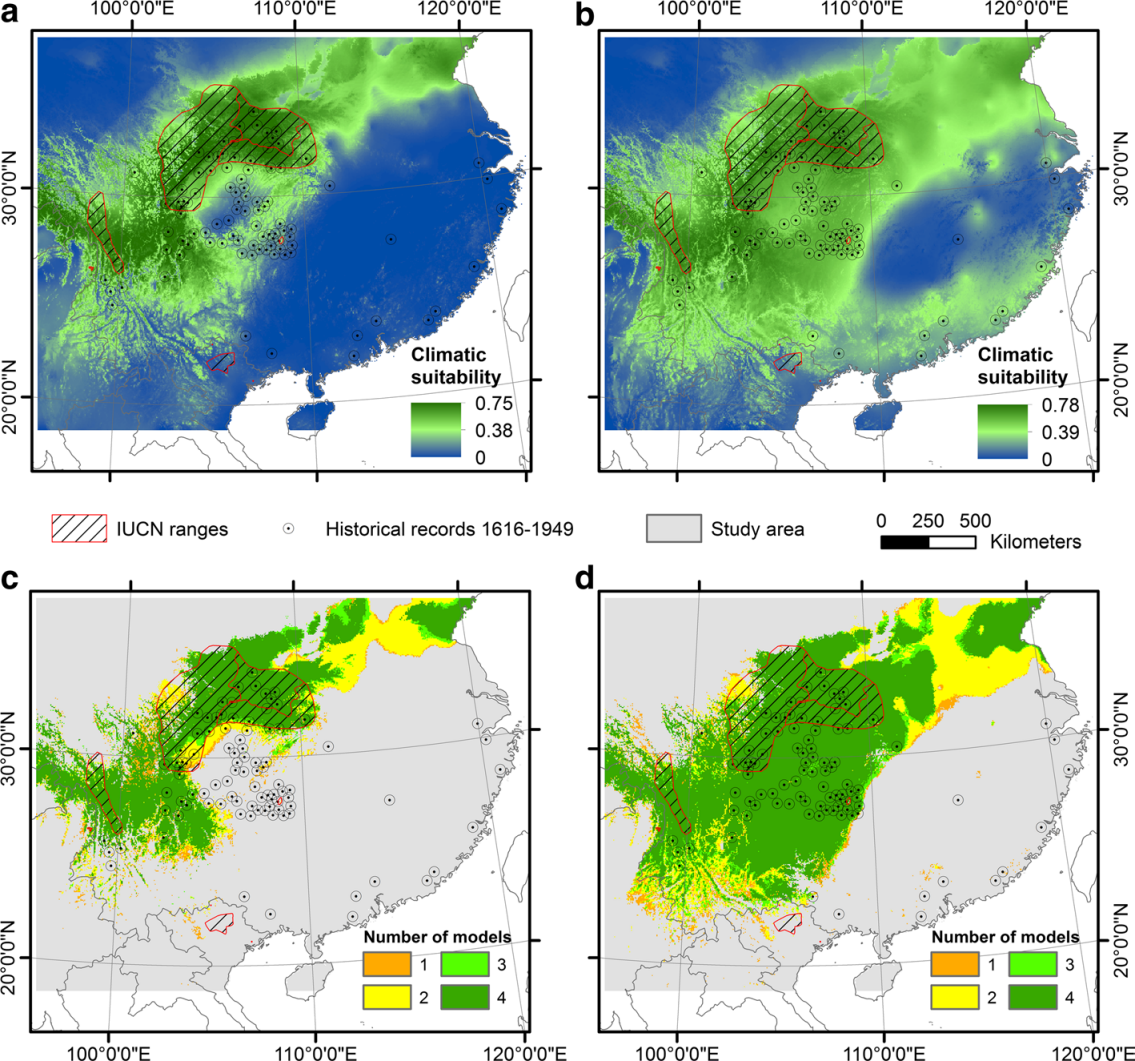
Mean climatic suitability and predicted climatic distribution using ensemble intersection and the 10th percentile training presence threshold for the 2 × 4 models (Table 1) at a 5 km × 5 km resolution. **a** Mean climatic suitability using only current range data and **b** using both current and historical data from 1616 to 1949. **c** Ensemble intersection for model CURR1–4 using only current range data and **d** for model HIST1–4 using both current and historical data from 1616 to 1949. Colours indicate number of models (ranging from 1 to 4) predicting values for climatic suitability above the 10th percentile training presence threshold. No colour within the study area indicates that none of the models predicted values for climatic suitability above the 10th percentile training presence threshold

**Fig. 6:**
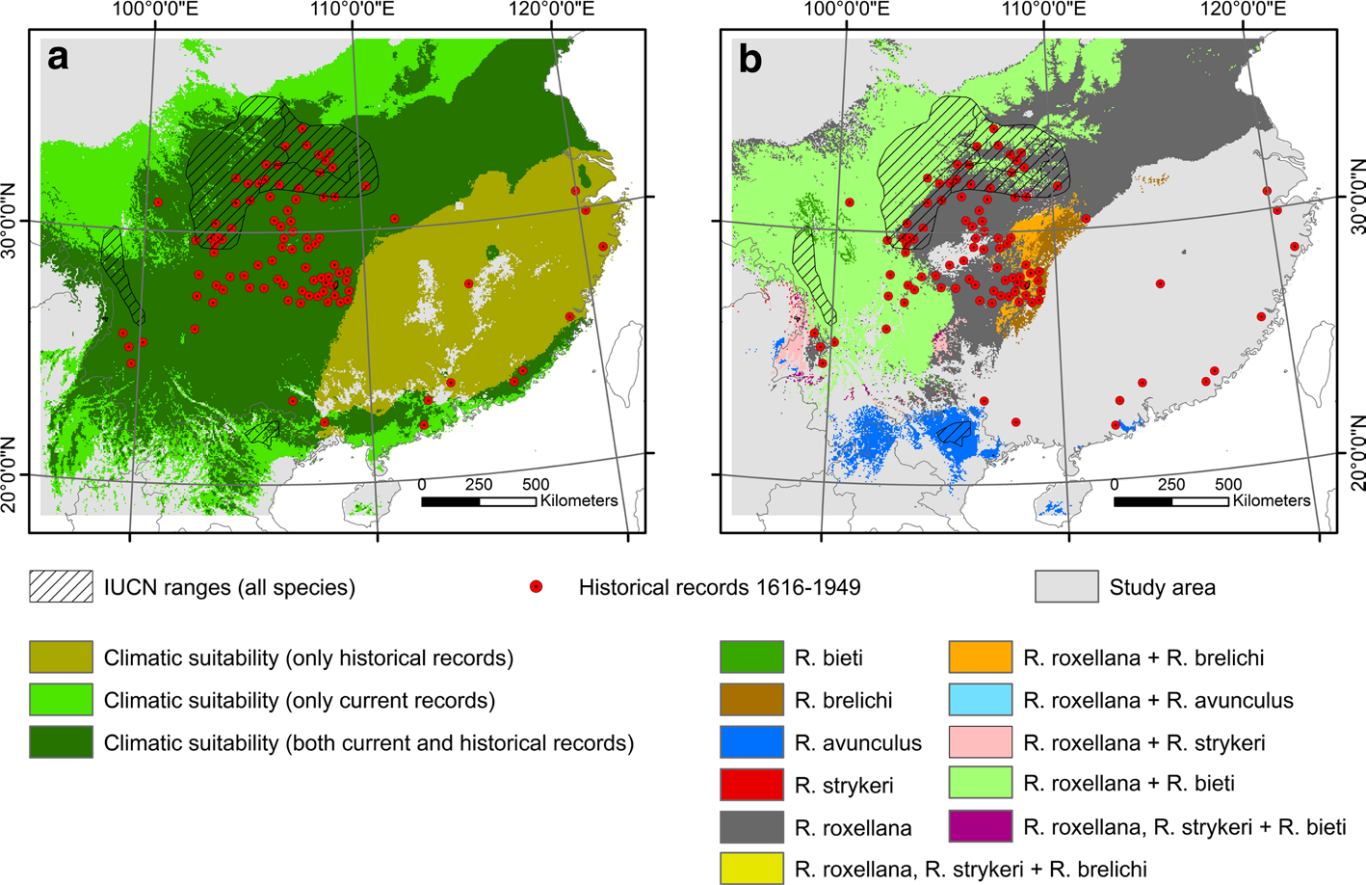
Climatic suitable area, defined as area within minimum and maximum values of the five climatic variables, PANN, PDryQ, MinTCM, MTCQ and MTWQ, at a 5 km × 5 km resolution. **a** Model for all five species together, using only current data from IUCN ranges (light and dark green) and area included if historical records is also used (brown green). Temperature data for historical records are adjusted with − 0.7 °C (see Supporting Information Appendix S7 for other climate adjustments). **b** Climatic suitable area, defined as area within minimum and maximum values of the five climatic variables, PANN, PDryQ, MinTCM, MTCQ and MTWQ, at a 5 km × 5 km resolution for each species separately and the overlap between them

The areas predicted as climatic suitable using Maxent were 72–89% larger when historical records were also included (Fig. 5 and Table 2). The same tendencies were also obtained using rectilinear bioclimatic envelope modelling for all species together and the historical records (Fig. 6). However, the area predicted climatic suitable by the historical records in the models changed to some degree depending on which down-adjusted climate data that were used. The more down-adjusted climate data that were used, the less areas were considered climatic suitable. Primarily areas in South and South-central China were affected by the adjusted climate data (see Table 2 and Supporting Information Appendix S7 and S8 for comparison of the predicted area with the different adjusted climate data).

**Table 2:** Area predicted (km^2^) by the Maxent models and the bioclimatic envelope models

Maxent models			Bioclimatic envelope with current and historical records						
Models	CURR1–4	HIST1–4	Climate adjusted^c^	Total area	Within PA	Within PA ≥ 50%	Within PA ≥ 75%	Outside PA ≥ 50%	Outside PA ≥ 75%
Min. one model^a^	1,112,500	1,914,200	0.0	3,716,625	319,325	100,275	17,550	824,425	62,100
			− 0.7	3,648,500	318,950	100,200	17,550	816,975	61,200
All four models^b^	705,275	1,336,300	− 1.5	3,420,100	313,100	98,600	17,300	774,675	58,575
			− 2.0	3,284,275	305,800	96,525	17,175	738,425	57,350

^a^Predicted by minimum one of the four models

^b^Overlap of predicted area of all four models, CURR1–4 and HIST1–4 respectively

^c^Climatic data used to model; 0.0 = current climate data without any adjustment, − 0.7, − 1.5, and − 2.0 = current climate data adjusted with − 0.7, − 1.5, and − 2.0 °C, respectively

**Fig. 7:**
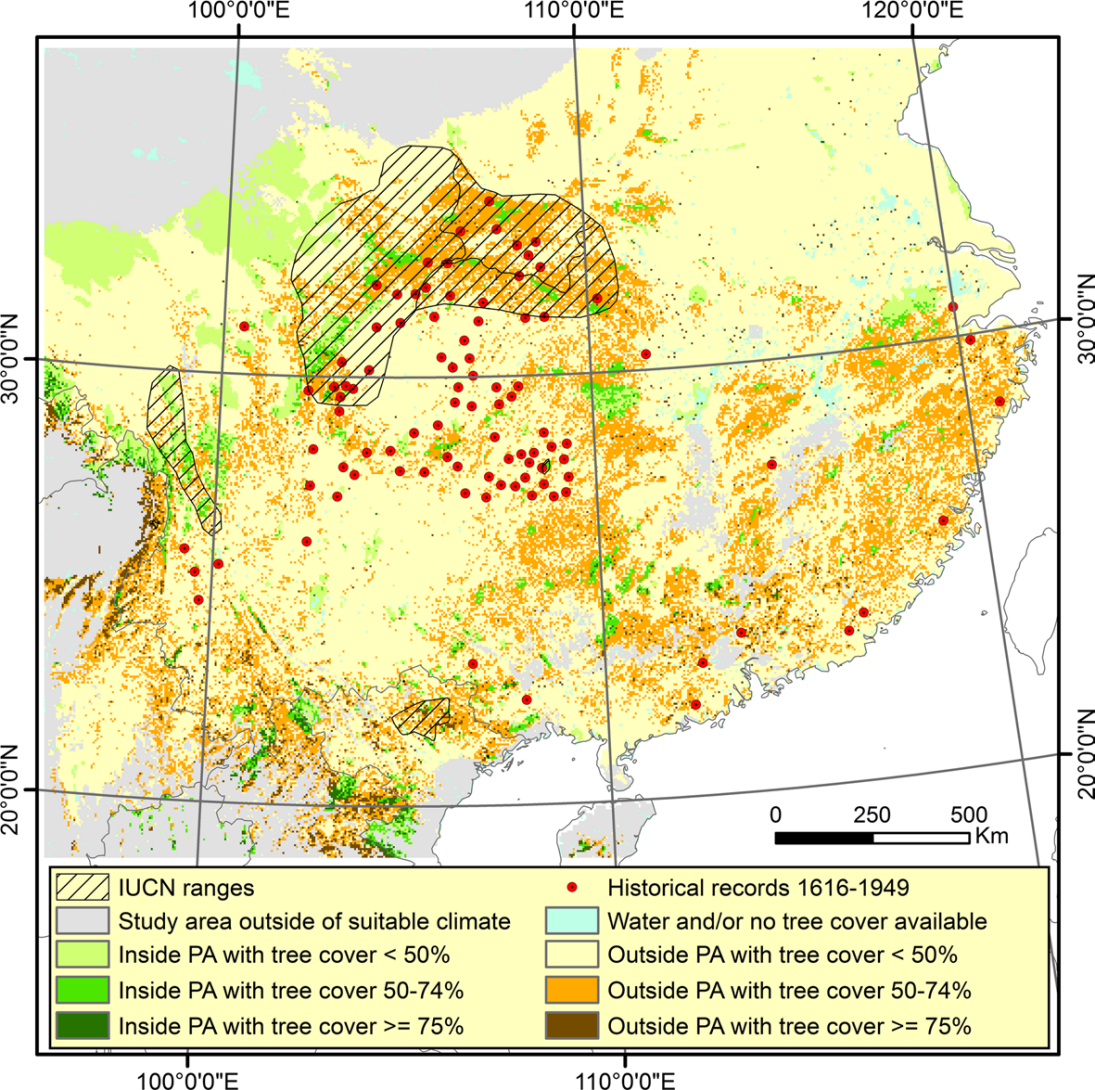
Climatic suitable area as defined in Fig. 6 outside protected areas (PA) (sand colors) and within protected areas (PA) (green colors). The darker sand or green color the higher tree cover percentage

The Maxent results were largely consistent with the rectilinear bioclimatic envelope models for the individual species (Fig. 6). The rectilinear bioclimatic envelope analyses at the genus level and including the historical records showed, depending on the historical climate adjustment used, that between 305,800 and 319,325 km^2^ of the climatic suitable area in China are within protected areas, of which 95,525–100,275 km^2^ has tree cover ≥ 50% and 17,1775–17550 km^2^ has ≥ 75% tree cover. In addition, there are between 738,425 and 824,425 km^2^ outside protected areas but with tree cover ≥ 50% and of these 57,350–62,100 km^2^ has tree cover ≥ 75% (Fig. 7 and Table 2, and Supporting Information Appendix S9).

## Discussion

One or more species of *Rhinopithecus* were recently more widely distributed in China. All species are today confined to fragmented areas with declining populations and are listed as endangered or critically endangered on the IUCN Red List (Bleisch et al. 2008; Bleisch and Richardson 2008; Xuan Canh et al. 2008; Yongcheng and Richardson 2008; Geissmann et al. 2012) and at least three of the species are, with total population sizes under 1000 individuals, close to extinction. In this study, we assessed three key questions for the conservation of *Rhinopithecus*: which climatic variables determine the current distribution of this genus, to what extent has the niche space of the genus been truncated due to anthropogenic activities, and what is the potential distribution of the genus, using species distribution modelling (SDM) with both current and historical distribution data. All the models scored AUC values between 0.793 and 0.900 and TSS values between 0.507 and 0.685, indicating good predictive power. PANN, MTCQ, MinTCM, PDryQ, and MTWQ were the variables that best explained the distribution of *Rhinopithecus.* There is a clear trend that the historical records generally are from areas that are warmer, wetter, and in lower elevation then the current distribution records (except the ones from *R. avunculus*), indicating extensive niche truncation. This is the case regardless of whether the current climate data were down-adjusted by − 0.7, − 1.5, or − 2.0 °C. As such, models that included historical records projected climatically suitable habitat to occupy a larger geographic extent than models calibrated only with IUCN range data. This is the case using Maxent as well as rectilinear bioclimatic envelope modelling with different adjusted climate data. Based on the later, 74,525–79,650 km^2^ of the climatically suitable area has tree cover ≥ 75% and could therefore constitute suitable habitat for *Rhinopithecus*, hereof 17,175–17,550 km^2^ within protected areas. All *Rhinopithecus* species inhabit primary forest and grid cells with tree cover ≥ 75% might constitute important potential habitat. In addition, an additional 834,950–924,700 km^2^ climatically suitable area has tree cover between 50 and 75%, hereof 96,525–100,275 km^2^ within protected areas. This area may also be suitable ecologically, as *Rhinopithecus* species have been observed spending a considerable time on the ground feeding on fallen nuts, ground plants etc. (Long et al. 1994; Xiang et al. 2007b, 2012; Grueter et al. 2009a).

The accuracy of the modelling depends on the accuracy of the distribution data. A problem with accuracy of the distribution data may occur as the IUCN distribution polygons (IUCN 2014) we used as a basis for our current records undoubtedly include areas that are not currently inhabited by *Rhinopithecus* and may include areas that are outside the current realized climatic niche of the species. However, we reduce the risk of including areas beyond the current realized niche by excluding grid cells that are outside of the known elevation range of the genus, have low tree cover, or have high human population densities or human influence. Another problem regarding the possible inclusion of climate outside the current realized climatic niche of the species in the analyses is that we modelled all species together. However, the historical records could not be identified into species level and we can also see in the rectilinear bioclimatic envelope models for the individual species that it does not affect the overall result as they are in accordance with the rectilinear bioclimatic envelope model of the genus. Additionally, at least some of the *Rhinopithecus* species live in a fairly wide range of climates and have high dietary plasticity (Long et al. 1994; Yiming 2006; Guo et al. 2007; Grueter et al. 2009a; Xiang et al. 2007b, 2012; Wong et al. 2013). Moreover, both the Maxent and the rectilinear bioclimatic envelope model is in accordance with the ecoregions, which distributions have been similar from the earliest historical records used (1616) to present (Ni et al. 2014) (see Supporting Information Appendix S1).

Another aspect worth discussing is that China experienced a cold period between 1321 and 1920 (Ge et al. 2013), which to some extent coincides with the disappearance of *Rhinopithecus* in southeast China. However, it does not make ecological sense that *Rhinopithecus* should retreat to higher elevations and hence even colder conditions during a period of increased cold. If temperature changes had affected its range dynamics in this period, *Rhinopithecus* would logically have been expected to retreat to warmer areas, or first retreat to the colder areas after 1920 when the current warm period began, i.e., both scenarios in contrast to the observed pattern. Furthermore, *Rhinopithecus* also lived in southeast China during the last warm period before 1321.

Our analyses strongly suggest that the *Rhinopithecus* species now survive as refugee species sensu Cromsigt et al. (2012). Comparing the SDM results with and without the historical records show that there clearly has been truncation of the occupied environmental space with respect to temperature and precipitation. The historical records are generally from warmer and wetter areas, and in lower elevation than the current distribution. This is also the reason why most of South, Southeast and Central China, which is warmer and wetter than the areas where the species is found today, only is predicted as climatic suitable if the historical records are included in the analyses. Hence, the reason why a large area in Southeast China is not predicted as suitable for *Rhinopithecus,* when only current distribution data is used, is mainly due to too high levels of precipitation, likely reflecting that high-rainfall areas have historically been more suitable for agriculture and human settlement, with the disappearance of *Rhinopithecus* from these areas synchronous with a rapid increase in human population and cultivated areas here during the last 400 years (Durand 1960; Li et al. 2002). In contrast, we are not aware of any plausible mechanisms whereby high rainfall would exclude *Rhinopithecus* directly or via non-anthropogenic indirect effects. Today, *Rhinopithecus* are largely restricted to higher elevations than much of their historical distribution. Li et al. (2002) provide evidence that *Rhinopithecus* was first extirpated from lower elevation during the last 400 years. Higher elevation areas are more remote and difficult for humans to access and utilize and other studies have found less deforestation, more reforestation and afforestation, less range contraction, and less extinction in topographically steep areas (Laliberte and Ripple 2004; Fisher 2011; Sandel and Svenning 2013; Faurby and Svenning 2015; Nüchel and Svenning 2017). Furthermore, many other species in the region, e.g., giant panda (*Ailuropoda melanoleuca*) and red panda (*Ailurus fulgens*), have also been impacted by strong anthropogenic pressure during the recent centuries with population declines and range retractions as consequences (Ceballos and Ehrlich 2002; Zhu et al. 2010; Hu et al. 2011). As such, it is likely that *Rhinopithecus* have been locally extirpated by anthropogenic factors, and are now restricted to areas with higher elevation and which are less accessible, even though these may be suboptimal habitats. Hence, *Rhinopithecus* is likely a refugee taxon, living in an anthropogenic truncated niche space.

Even though our results show that there are large areas that are climatic suitable for the genus, have tree cover between 50 and 75% or above 75%, and some even protected, it does not mean that the species can actually live in these areas. For example, our modelling does not show whether or not the vegetation meets the different *Rhinopithecus* species’ specific requirements in terms of food and habitat structure. Today, *R. avunculus* is found in tropical evergreen (Xuan Canh et al. 2008). *Rhinopithecus brelichi* is found in mixed deciduous and evergreen broadleaf (Bleisch et al. 2008; Xiang et al. 2009)*. Rhinopithecus roxellana* and *R. strykeri* is found in mixed deciduous and evergreen broadleaf and mixed conifer-broadleaf forest (Guo et al. 2007; Yongcheng and Richardson 2008; Chi et al. 2014), and *R. bieti* is found in high-altitude evergreen forest (Long et al. 1994; Bleisch and Richardson 2008; Xiang et al. 2011). They are all folivorous, but also feed on fruits, seeds, insects and lichens is also an important part of the diet for *R. roxellana* and especially *R. bieti* (Kirkpatrick 1995; Quyet et al. 2007; Guo et al. 2007; Xuan Canh et al. 2008; Grueter et al. 2009a, b; Xiang et al. 2012). In addition, some if not many of the protected areas might be so-called “paper parks”, where no or little management and law enforcement is done (Harkness 1998; Joppa et al. 2008), meaning that they offer little to no real protection. This is particularly a problem in relation to that poaching and illegal timber extraction threatens many of the extant populations (Xiao et al. 2003; Xiang et al. 2007a, 2009; Hoang 2010). Moreover, many areas have high anthropogenic pressure, e.g., in terms of human populations densities, agriculture and many suitable areas will be unreachable for *Rhinopithecus* due to natural and anthropogenic dispersal barriers such as rivers, urban areas and agricultural land (Liu et al. 2009; Chang et al. 2012). Other studies which have tried to model the potential distribution of species have also emphasised the importance of taking anthropogenic pressure into account (Kuemmerle et al. 2011; Escobar et al. 2015). In our study, human population density contributed to the predictive power of our models, while HII contributed little. The reason why HII has little effect on our models more is likely due to the coarse scale of our data but also the fact that there indeed is high anthropogenic pressure within some of the species current ranges (Xiang et al. 2009). In addition, the large reforestation and afforestation programs in China, might offer great opportunities in connection with the above mentioned concerns about habitat requirements, dispersal barriers and anthropogenic pressure (Yin et al. 2005).

SDMs are frequently used to assess which variables influence the distribution of a taxa, and to identify its potential distribution. This makes SDMs particularly useful for conservation management. However, SDMs often rest on the assumption that the current realized niche more or less represents the fundamental niche. Many species have been locally extirpated by anthropogenic pressure and if SDMs does not take this into account and only use current distribution data to model the potential distribution they might not show the “true picture” of a species tolerance to e.g. climate. Our models are coarse, but like other studies, e.g. Chatterjee et al. (2012), they emphasise the importance in the use of historical records/distribution data for mapping the potential distribution of species. This is especially of the utmost importance in conservation studies, where e.g. the influence from climate change and reintroduction potential is being modelled for threatened species, which might be refugee species living in an anthropogenically truncated niche space.

Much is still not known about the ecology of *Rhinopithecus* and as stated in the introduction they face an uncertain future. To enhance the survival prospects of *Rhinopithecus*, populations and distributions should be increased, and it is of utmost importance to learn more about their ecology and habitat requirements, protect existing and potential habitat, make habitat corridors and investigate the possibilities for assisted migration. Our results show that there is likely much potential for expanding some of the *Rhinopithecus* species’ distribution to parts of the former range of the genus, and they can be used as a basis to further investigate which areas that might be used for reintroduction of *Rhinopithecus* or to connect existing populations through habitat corridors.

## Electronic supplementary material

Below is the link to the electronic supplementary material.

Supplementary material 1 (TIFF 981 kb). Map of terrestrial ecoregions (vegetation zones) derived from Olson et al. 2001

Supplementary material 2 (EPS 2070 kb) Pairwise Pearson’s correlations coefficient (r) for the eight climatic variables

Supplementary material 3 (AI 178 kb) a) Results of jackknife evaluation of the relative importance of the variables with respect to regularized training gain for model CURR1 with anthropogenic variables (HII and Pop10). For acronyms see the environmental data section in methods. b) Estimated response curves (logistic output: probability of presence) for Human Influence Index (HII) and human population density for 2010 (Pop10), which show how the logistic prediction changes as each environmental variable is varied, keeping all other variables at their average sample value, for model CURR1 with HII and Pop10

Supplementary material 4 (TIFF 11477 kb). Mean suitability for Maxent models at a 5 km × 5 km resolution using current distribution data and the variables PDryQ, PANN, MTCQ, MTWQ, HII, and Pop10

Supplementary material 5 (TIFF 2363 kb). Comparison of thresholds. Values for climatic suitability above different selected threshold, for model HIST1

Supplementary material 6 (EPS 6968 kb). Boxplot of minimum temperature of coldest month (MinTCM), mean temperature of warmest quarter (MTWQ) and annual precipitation (PANN) for the historical records, the IUCN ranges (all five species together) and for the five species separately. MinTCM and MTWQ for the historical records are divided into four categories; current climate data without adjustments, current climate data adjusted with − 0.7 °C, − 1.5 °C, and − 2.0 °C, respectively

Supplementary material 7 (TIFF 3259 kb). Climatic sensitivity analysis. Comparison of the effect of adjusted climate variables on the climatic suitable area, defined as area within minimum and maximum values of the five climatic variables, PANN, PDryQ, MinTCM, MTCQ and MTWQ model for all five species together, at a 5 km × 5 km resolution. Temperature data for historical records is adjusted with a) 0.0 °C, b), − 0.7 °C, c) − 1.5 °C, and d) − 2.0 °C

Supplementary material 8 (TIFF 2817 kb). Climatic sensitivity analysis. Comparison of the effect of adjusted climate variables on the climatic suitable area, defined as area within minimum and maximum values of annual mean temperature (AMT) model for all five species together, at a 5 km × 5 km resolution. Temperature data for historical records is adjusted with a) 0.0 °C, b), − 0.7 °C, c) − 1.5 °C, and d) − 2.0 °C

Supplementary material 9 (TIFF 4577 kb). Climatic suitable area as defined in S7 for the different adjusted climate, a) 0.0 °C, b), − 0.7 °C, c) − 1.5 °C, and d) − 2.0 °C. Sand colors indicate outside protected areas (PA) and green colors indicate within protected areas (PA). The darker sand or green color the higher tree cover percentage
