# Supplementary figures and images for: Snub-nosed monkeys (Rhinopithecus): potential distribution and its implication for conservation
Source: Biodivers Conserv. 2018 Jan 23;27(6):1517–38. doi: 10.1007/s10531-018-1507-0 (PMC6560942; doi:10.1007/s10531-018-1507-0)

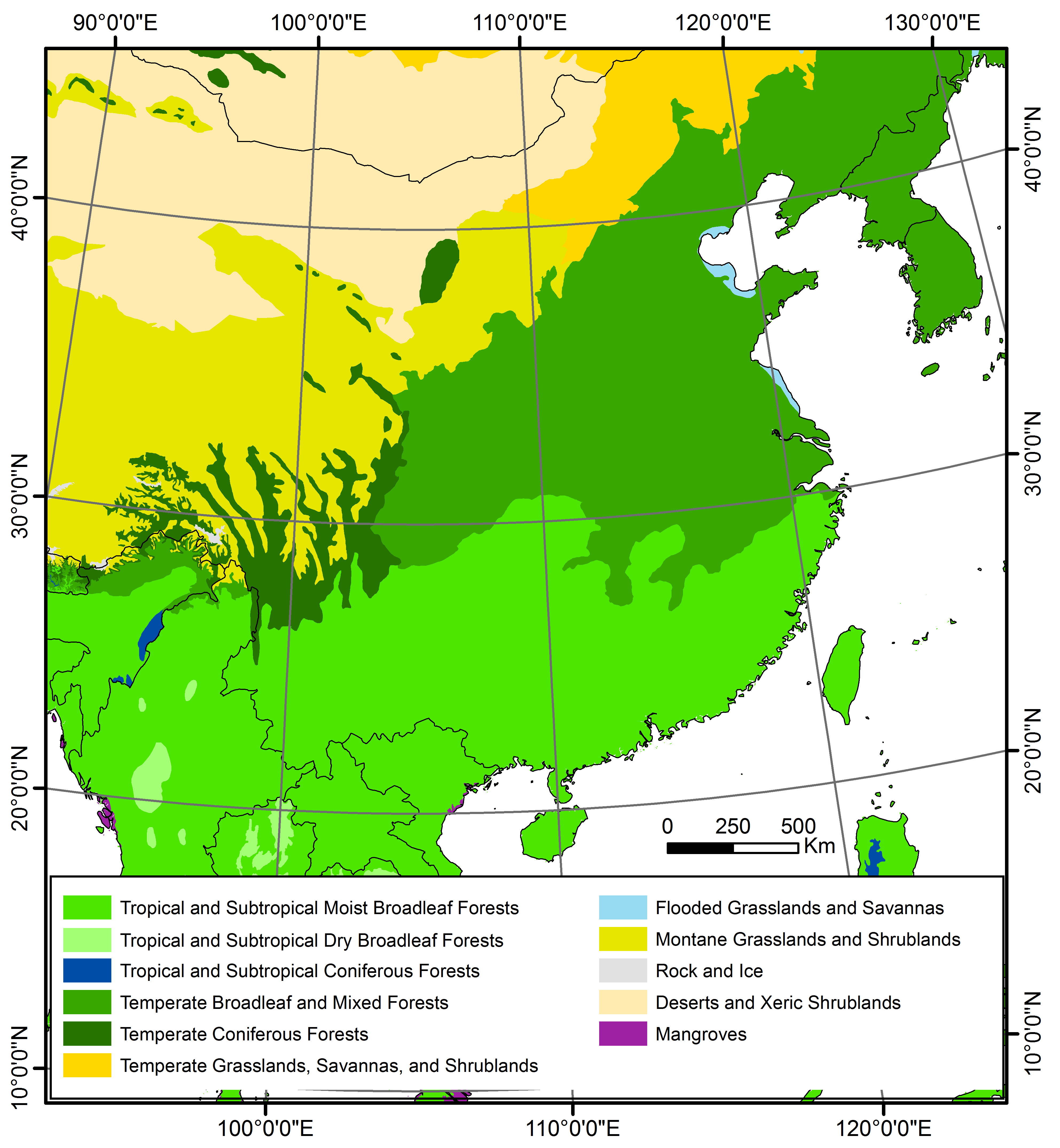

Supplement: Supplementary file 1 — Supplementary material 1 (TIFF 981 kb). Map of terrestrial ecoregions (vegetation zones) derived from Olson et al. 2001 [file 10531_2018_1507_MOESM1_ESM.tif]

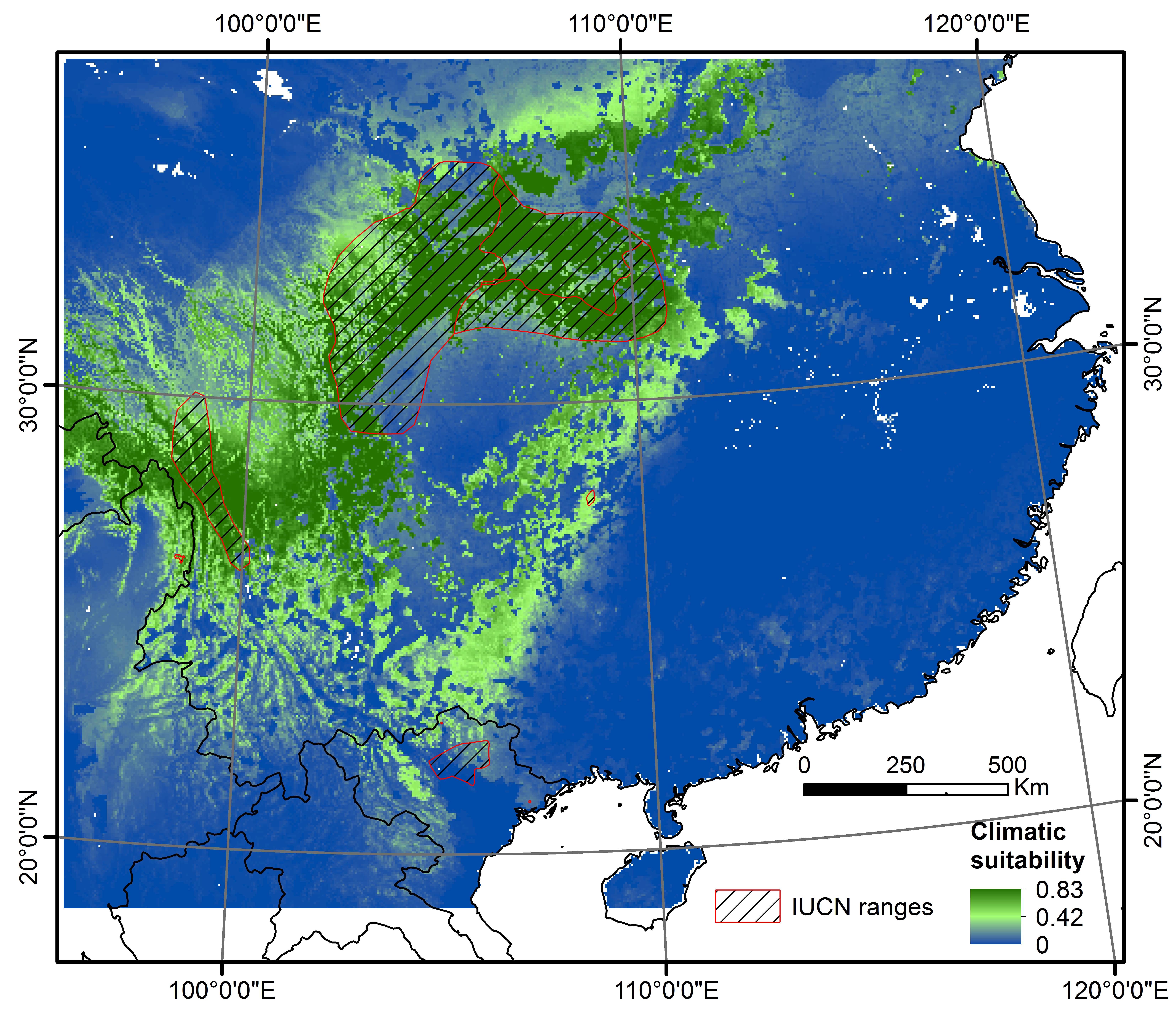

Supplement: Supplementary file 4 — Supplementary material 4 (TIFF 11477 kb). Mean suitability for Maxent models at a 5 km × 5 km resolution using current distribution data and the variables PDryQ, PANN, MTCQ, MTWQ, HII, and Pop10 [file 10531_2018_1507_MOESM4_ESM.tif]

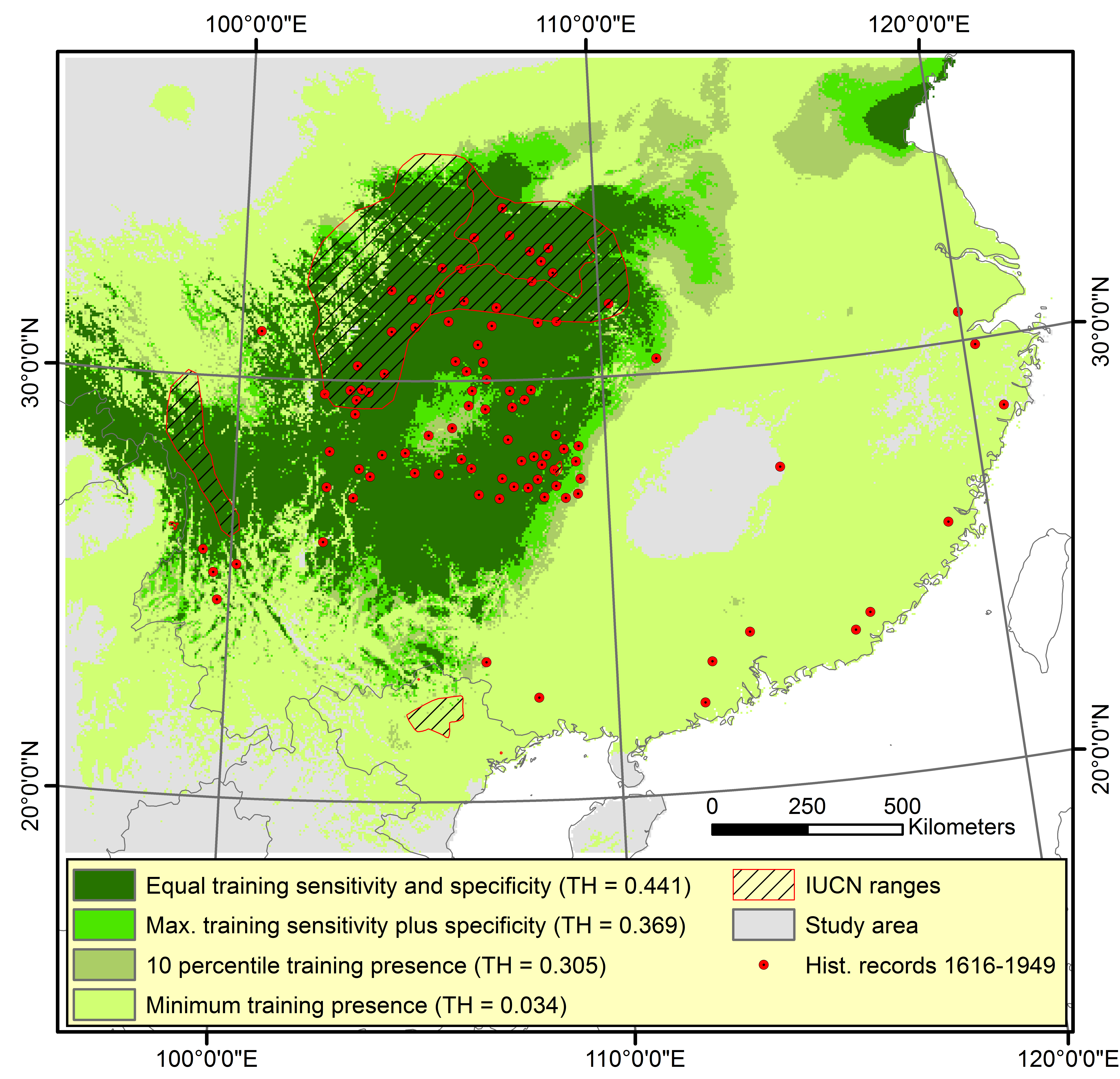

Supplement: Supplementary file 5 — Supplementary material 5 (TIFF 2363 kb). Comparison of thresholds. Values for climatic suitability above different selected threshold, for model HIST1 [file 10531_2018_1507_MOESM5_ESM.tif]
